# A 6-Month Follow-Up Study on Worry and Its Impact on Well-Being During the First Wave of COVID-19 Pandemic in an Italian Sample

**DOI:** 10.3389/fpsyg.2021.703214

**Published:** 2021-10-13

**Authors:** Giulia Ongaro, Clizia Cincidda, Valeria Sebri, Lucrezia Savioni, Stefano Triberti, Roberta Ferrucci, Barbara Poletti, Bernardo Dell’Osso, Gabriella Pravettoni

**Affiliations:** ^1^Applied Research Division for Cognitive and Psychological Science, IEO, European Institute of Oncology IRCCS, Milan, Italy; ^2^Department of Oncology and Hemato-Oncology, University of Milan, Milan, Italy; ^3^Aldo Ravelli Center for Neurotechnology and Experimental Brain Therapeutics, Department of Health Sciences, University of Milan, Milan, Italy; ^4^Neurology Clinic III, ASST Santi Paolo e Carlo, Milan, Italy; ^5^Istituto di Ricovero e Cura a Carattere Scientifico (IRCCS) Ca’ Granda Foundation Maggiore Policlinico Hospital, Milan, Italy; ^6^Department of Neurology and Laboratory of Neuroscience, Istituto Auxologico Italiano, Istituto di Ricovero e Cura a Carattere Scientifico (IRCCS), Milan, Italy; ^7^Department of Biomedical and Clinical Sciences Luigi Sacco, Department of Mental Health, University of Milan, ASST Fatebenefratelli-Sacco, Milan, Italy; ^8^CRC Aldo Ravelli, Milan, Italy; ^9^Department of Psychiatry and Behavioural Sciences, Stanford University, Stanford, CA, United States

**Keywords:** COVID-19, psychological well-being, worry, longitudinal study, personality traits

## Abstract

The Italian state adopted serious safety measures to manage the COVID-19 pandemic in the year 2020. The lockdown was associated with negative psychological consequences in healthy populations, mostly in terms of anxiety, distress, depression, and even traumatic symptoms. This longitudinal study aimed at briefly documenting the psychological impact among an Italian sample, in terms of worry and its impact on psychological well-being levels, of the first wave of COVID-19, taking into account the changes in the lockdown scenario. A three-time follow-up survey was administered to 177 subjects (*Female*: 78%, *M*_*age*_ = 36.33), during (T0), at the end (T1), and 3 months after the end of the first lockdown (T2). Since the first wave of COVID-19, results showed a decrease in worry and the perception of virus diffusion’s controllability over time while psychological well-being increased. Furthermore, factors such as personality traits (neuroticism and agreeableness) and dysfunctional coping strategies predicted increases in worry levels at the end of the lockdown and 3 months after in the Italian context. However, worry levels during and at the end of the lockdown did not predict well-being levels 3 months after the end of the lockdown. Based on these findings, mental health policymakers should design tailored interventions able to improve the perception of virus diffusion management, as well as address the psychological needs of Italian citizens and support it, including a plan for the follow-up evaluation.

## Introduction

The Italian state adopted serious safety measures to manage the COVID-19 pandemic in the year 2020 (Vicentini et al., 2020). Starting from March 11, an extensive lockdown was adopted, featuring the closure of commercial activities, schools, and the cancelation of public events. Citizens were requested to stay at home and to avoid social contact except for documented work or health emergency reasons. This severe lockdown (T0 for the sake of the present study) lasted until mid-May when most restrictions to the personal movement were mitigated (T1), and mid-September when they were terminated and the government announced that the “first wave” of COVID-19 had ended (T2). Nowadays, the pandemic health emergency is still ongoing along with intermittent lockdowns and limitations; thus, it is important to analyze citizens’ psychological state longitudinally.

Indeed the COVID-19 pandemic and related lockdowns brought a huge number of psychological and sociological studies to account for international citizens’ experience. The limitations imposed to work and movement improved notable economic losses (Cerami et al., 2020; Codagnone et al., 2020) and the lockdown triggered negative psychological consequences in healthy populations, mostly in terms of anxiety and distress (Castelli et al., 2020; Rossi et al., 2020; Cincidda et al., 2021; Petrocchi et al., 2021), depression (Meda et al., 2021) and even traumatic symptoms (Johnson et al., 2020; Masiero et al., 2020; Rossi et al., 2021) creating a burden that mental health services are likely to deal with for a long time (D’Agostino et al., 2020; Lasalvia et al., 2021). Qualitative research based on the critical incident technique (Durosini et al., 2021) showed that healthy citizens were able to experience also positive events during the lockdown (e.g., in terms of cultivation and enjoinment of relationships with loved ones), but at the same time they were subjected to notable emotional distress: for example, the daily experience of the lockdown, along with the alarming messages coming from the media and the unreal perception of emptiness and isolation in the cities, were connected to a novel “sensation of emergency” accompanied by everlasting negative arousal. Likewise, research showed that people with higher perception of COVID-19 severity and lower perception of control over the possibility of infection reported higher levels of worry and anxiety (Sebri et al., 2021). A longitudinal study (Pellerin and Raufaste, 2020) demonstrated how psychological resources created a buffer against the negative effects on well-being. In particular, the study highlighted how emotional well-being was positively predicted by gratitude and hope, and, to a lesser extent, by acceptance and how psychological well-being was positively predicted by wisdom, self-efficacy, and gratitude.

During COVID-19 pandemic, personality traits and in particular neuroticism emerged as one of the correlates of most of psychopathological outcomes and distress, although not as uniformly as expected (Kroencke et al., 2020; Lee and Crunk, 2020; Modersitzki et al., 2020; Somma et al., 2020). Unfortunately, very few studies investigated the association between personality traits and adjustment to COVID-19 with a longitudinal methodology (Rettew et al., 2021; Zacher and Rudolph, 2021). In these studies, authors revealed that higher levels of neuroticism favored increases in distress (Rettew et al., 2021; Zacher and Rudolph, 2021); higher levels in emotional stability anticipated decreases in perceived stressfulness of the COVID-19 pandemic (Zacher and Rudolph, 2021); while higher levels in agreeableness and conscientiousness anticipated increases in mood (Rettew et al., 2021).

The goal of the present contribution is to extend the information on the “tracking” of worry and emotional well-being of Italian citizens over the first wave of the COVID-19 pandemic, taking into account the changes in the lockdown scenario (T0, T1, T2). Specifically, it was hypothesized that:

• Hp1: worry levels will reduce from T0 to T2 while psychological well-being will increase from T0 to T2 considering that safety measures were increasingly mitigated;
• Hp2: higher worry scores at T0 and T1 will predict a decrease in psychological well-being at T2.

Also, explorative research questions are considered:

1. RQ1: which personality characteristics and individual coping strategies at T0 will contribute to predict worry scores at T2?
2. RQ2: which specific COVID-related worry affects people with high worry levels in the three evaluation times and which specific COVID-related worry affects at T2 people with specific personality traits?

## Methods

### Participants

759 respondents (out of a total sample of 1233) of the initial survey (Sebri et al., 2021) expressed their consent to participate in a follow-up study, gave email addresses, and were contacted to fulfill the next phase. 436 agreed to take part and complete the first follow-up evaluation but only 177 completed both the first (T1) and the second follow-up evaluation (T2). Thus, the final sample of this longitudinal study comprised 177 participants (Male: 39, 22%; Female: 138, 78%) that were included in the analysis. The mean age was 36.33 (SD 11.60), ranging from 20 to 69 years old. The majority of the sample was composed of adults, well-educated, white-collar workers, from the Northern regions of Italy, and lived with partners and/or children. Regarding the working status, the majority of first survey respondents were working from home (37.9%; *N* = 67) or continued working in presence (18.1%, *N* = 32). Other participants were students (4.5%, *N* = 8), unemployed (12.4%, *N* = 22), or in other working conditions (15.8%, *N* = 28). Three months after the end of the lockdown the majority of the participants returned to work under normal conditions (54.8%, *N* = 97). During all the evaluation, most of the participants were not infected by COVID-19 (T0: 99.4%; T1: 92.1%; T2: 94.9%), as for their acquaintances (T0: 98.9%; T1: 89.8%; T2: 82.8%). However, at the two follow-ups, respectively 7.3 and 3.8% of the participants showed symptoms similar to COVID-19 symptoms. More descriptive statistics are presented in Table 1.

**TABLE 1:** Socio-demographic characteristics of the study sample.

Sample (*N* = 177)	
**Age** (M ± SD, range)	36.33 ± 11.60; 20–69
**Age groups**	
Emerging adults	42.4% (75)
Adults	57.6% (102)
**Gender**	
Male	22% (39)
Female	78% (138)
**Educational level**	
Primary/Middle school	1.7% (3)
High school	18.6% (33)
Bachelor/Master’s degree	59.9% (106)
Post Ph.D.	19.8% (35)
**Employment**	
Student	9.6% (17)
Unemployed	4.5% (8)
Healthcare professional	4% (7)
Blue-collar	39% (69)
White-collar	42.9% (76)
**Provenience**	
North of Italy	70.6% (125)
Center of Italy	20.9% (37)
South of Italy	8.5% (15)
**Living with**	
No one	10.7% (19)
Family	29.4% (52)
Partner and/or children	55.4% (98)
Roommates	4.5% (8)

### Materials and Procedure

The current study was approved by the lead author’s Institutional Review Board (IRB), in conformity with the principles embodied in the Declaration of Helsinki. An anonymous online survey was set on Qualtrics and distributed on various internet platforms to evaluate worry and psychological well-being in an Italian sample during COVID-19 lockdown, 2 and 6 months after it began. Specifically, the survey was administered on the same online platform as the baseline (Sebri et al., 2021). Data were collected at the baseline (T0) from March 20 to April 10, 2020, after 2 months (T1) from May 15 to May 30, 2020 and after 6 months (T2) from 15 September to 30 September, 2020. A self-administered questionnaire was created to assess socio-demographic characteristics such as biological sex, age, education, provenience, employment, and living conditions during the COVID-19 outbreak. Furthermore, information related to COVID-19 was collected and participants were asked to indicate their working status and if they, their acquaintances, or loved ones (such as family members or friends) were infected with COVID-19 in all three times of evaluation. Moreover, two *ad hoc* questionnaires had been administered in the three surveys:

• *COVID-19 severity and controllability:* we assessed the individual perception of COVID-19 severity in terms of mortality, rate, morbidity, and the current impact on both social and economic aspects in Italy with 5 items on a 5-point Likert scale (ranging from “not severe at all” to “very severe”). Participants’ perception of controllability over the possibility to contract or spread COVID-19 infection was evaluated with two items on a 5-point Likert scale (ranging from “totally uncontrollable” to “totally controllable”).
• *COVID-19 related worry:* worry levels were assessed regarding some key areas: economic impact of the pandemic and lockdown; the challenge of recovering the previous lifestyle; the risks inherent to meeting unknown people; changes in future life plans; the risk of personally contracting COVID-19; the risk of significant others contracting COVID-19; and the recurrence of the health emergency in the future. All of these sources of worry were assessed with one specific item on a 3-point Likert scale, ranging from “not worried” to “very worried.”

Psychological well-being and coping strategies that resulted associated with worry levels during the COVID-19 crisis in the first evaluation (Sebri et al., 2021) were evaluated also in both the follow-up administrations, using the same self-report and standardized questionnaires at all times (T0, T1 and T2): Psychological General Well Being Index (PGWBI—Grossi et al., 2006) to measure psychological distress and affective well-being (note that this measure names “distress” the low levels of well-being, although this aspect is controversial; Winefield et al., 2012)., that are defined as the reactions to internal and external demands characterized by heterogeneous psychological symptoms, such as low self-esteem, hopelessness, sadness, helplessness, and fear (Dohrenwend et al., 1980), and the prevalence of positive affect over negative affect, respectively (Kahneman et al., 1999); Brief Coping Orientation to Problems Experienced Inventory (Brief-COPE—Monzani et al., 2015) based on the assessment of coping strategies recognizing as thoughts and behaviors that individuals use to manage the internal and external demands of situations that are appraised as stressful (Lazarus and Folkman, 1984); and Penn State Worry Questionnaire (PSWQ- Morani et al., 1999) that measures the intensity of worry, a sequence of uncontrolled thoughts that may evoke elevated levels of anxiety and distress closely related to the fear of uncertain and probably negative outcomes (Kelly and Miller, 1999). We then supplemented this survey data with previously collected data on personality traits, such as the relatively enduring patterns of thoughts, behaviors, and feelings that distinguish an individual from another one (Roberts et al., 2008), evaluated using Big Five Inventory–Short Form (BFI-S—Guido et al., 2015). Specific characteristics of these scales have been largely explained in the first phase of our study (Sebri et al., 2021).

### Data Analysis

Data analyses were performed using the statistical software analysis package SPSS (Version 26.0). First, within-subjects ANOVA analysis was run to explore the differences in the mean scores of the psychological variables (PSWQ, PGWBI, Controllability of virus diffusion) over the three times of evaluation. Second, stepwise multivariable regression analyses were run to investigate the association between PSWQ scores during the final period of the lockdown, controlling for demographic variables and PSWQ scores at the baseline, and the following independent variables: Brief COPE and BFI-S scores from the initial period of the lockdown, because they resulted to be significant predictors in the first period of the lockdown (Sebri et al., 2021). The collinearity assumption was checked before running the model. The threshold level of statistical significance for each variable to enter the model set was *p* < 0.05.

## Results

### Differences Between the Initial, the Final Period of the Lockdown and After 3 Months

Table 2 reports the average scores on the psychological variables over the three times of the study. The results of the within-subjects ANOVA revealed a significant difference between the baseline and the final evaluation (T2). In particular, worry decreased significantly over time [*F*_(2,278)_ = 42.96, *p <* 0.001, $\eta_{p}^{2}$ = 0.24], and *post hoc* pairwise comparisons showed that worry is reduced by 6.586 between T0 and T2 (*p* < 0.001) and it is reduced by 5.379 between T1 and T2 (*p* < 0.001). Instead psychological well-being increased significantly [*F*_(2,280)_ = 9.97, *p* < 0.001, $\eta_{p}^{2}$ = 0.06], specifically between T0 and T1 (*p* < 0.001) and between T1 and T2 (*p* < 0.001), as shown by *post hoc* pairwise comparisons. The perception of controllability of virus diffusion significantly decreased between the lockdown phase and the end of the first wave of COVID-19 pandemic [*F*_(2,312)_ = 10.70, *p* < 0.001, $\eta_{p}^{2}$ = 0.06]. *Post hoc* pairwise comparisons showed that the perception of controllability reduced by.204 between T0 and T1 (*p* < 0.001) and then reduced by an additional.146 between T1 and T2 (*p* = 0.004).

**TABLE 2:** Difference between evaluation times in levels of worry, psychological well-being and controllability of COVID-19 diffusion.

Variables	*M* (*SD*) T0	*M* (*SD*) T1	*M* (*SD*) T2
PSWQ	44.04 ± 11.89	42.84 ± 11.78	37.46 ± 7.57
PGWBI	74.67 ± 14.22	78.12 ± 14.28	78.89 ± 14.07
Controllability of virus diffusion	4.3 ± 0.76	4.11 ± 0.81	3.96 ± 0.85

*PSWQ, Penn State Worry Questionnaire; PGWBI, Psychological General Well-Being Index.*

### Regression Analysis

Based on our hypothesis, a linear regression analysis was run to verify whether worry levels collected during the initial period of the lockdown (T0) and at the end of the lockdown (T1) predicted the level of psychological general well-being 3 months after the end of lockdown (T2), controlling for T0 and T1 well-being levels and socio demographic information (gender and age). Worry levels at T0 and T1 showed a significant negative correlation with psychological well-being levels at T2 (T0: *r* = −0.46; *p* < 0.01; T1: *r* = −0.58; *p* < 0.01). For model 1, we entered worry and well-being levels at T0 controlling for socio-demographic information, and then we also entered worry and well-being levels at T1. The final model was significant [*F*_(2,138)_ = 72.379, *p* < 0.001], and explained 51.2% of variance in the level of psychological well-being at T2 [*R*^2^ = 0.512, Adjusted *R*^2^ = 0.505, Δ*F*_(1,138)_ = 48.39, *p* < 0.001]. The results of the regression indicated that only well-being level at T0 and at T1 predicted levels of psychological general well-being 3 months after the end of the lockdown (PGWBI_T0: β = 0.225, *p* < 0.01; PGWBI_T1: β = 0.547, *p* < 0.001). However, PSWQ scores collected at T0 and T1 were excluded from the model. Table 3 showed results of regression analysis on psychological well-being 3 months after the end of the lockdown.

**TABLE 3:** Stepwise regression analysis on worry and psychological well-being during COVID-19.

Outcome	Predictors	β	*t*	*R* ^2^	*F*	ΔR	ΔF
PGWBI_T2	Model 1						
	PGWBI_T0	0.584	8.477***	0.341	71.866***		
	Model 2						
	PGWBI_T0	0.225	2.861**				
	PGWBI_T1	0.547	6.956***	0.512	72.370***	0.171	48.390***
PWSQ_ T2	Model 1						
	PSWQ_T1	0.676	10.767***	0.457	115.930***		
	Model 2						
	PSWQ_T1	0.512	5.617***				
	PSWQ_T0	0.222	2.437*	0.472	63.009***	0.023	5.939*

*p < 0.05; **p < 0.01; ***p < 0.001.

*PGWB, Psychological General Well-Being Index; PSWQ, Penn State Worry Questionnaire.*

In order to fully test Hp2 and to analyze the direction of the association between worry and psychological well-being, a linear regression analysis was run to verify whether well-being levels collected during the initial period of the lockdown (T0) and at the end of the lockdown (T1) predicted the level of worry 3 months after the end of lockdown (T2), controlling for T0 and T1 worry levels and socio demographic information (gender and age). The model was significant [*F*_(2,137)_ = 63.009, *p* < 0.001] but the results of the regression indicated that only worry levels at T0 and at T1 predicted worry levels at T2. PGWBI scores collected at T0 and T1 were excluded from the model.

In order to answer to RQ1, two stepwise multiple regression analyses were run, including as predictors the psychological variables (personality traits and coping strategies) that prior research has shown impacting worry levels during the first period of the lockdown. The Brief COPE, the Big Five-S, the psychological well-being and PSWQ scores, collected during the initial period of the lockdown (T0), were included as predictors. PSWQ scores, collected both at the end of the lockdown (T1) and 3 months after the end of the lockdown (T2), were considered as an outcome. Both stepwise multiple regression analyses were controlled for demographic characteristics (gender and age). Table 4 shows the results of the multiple regressions analysis.

**TABLE 4:** Stepwise regression analysis on worry during COVID-19 with personality traits and coping strategies as predictors.

Outcome	Predictors	β	*t*	*R* ^2^	*F*	Δ R	Δ F
PSWQ_ T1	Model 1						
	PSWQ_T0	0.767	15.792***	0.589	249.37***		
	Model 2						
	PSWQ_T0	0.597	8.823***				
	Neuroticism	0.237	3.508***	0.616	138.944***	0.027	12.308***
PSWQ_ T2	Model 1						
	PSWQ_T0	0.599	8.795***	0.359	77.347***		
	Model 2						
	PSWQ_T0	0.510	7.199***				
	Dysfunctional coping	0.239	3.378***	0.408	47.297***	0.049	11.412***
	Model 3						
	PSWQ_T0	0.470	6.559***				
	Dysfunctional coping	0.247	3.533***				
	Agreeableness	−0.156	−2.334*	0.431	34.372***	0.023	5.450*

*p < 0.05; ***p < 0.001.

*PSWQ, Penn State Worry Questionnaire.*

In the first analysis, we inserted the PSWQ scores collected at the end of the lockdown (T1) as the outcome. The final model, that included neuroticism, and worry levels during the lockdown as predictor, accounted for a significant proportion of the variance in level of worry [*R*^2^ = 0.616, Adjusted *R*^2^ = 0.612, Δ*F*_(1,173)_ = 12.308, *p* = 0.001]. Specifically, initial higher neuroticism levels predicted increases in worry from T0 to T1, after controlling for initial worry levels and socio-demographic data (*Neuroticism*: β = 0.237, *p* < 0.001). Extraversion, agreeableness, openness, conscientiousness, emotion- and problem-focused coping, dysfunctional coping, and psychological well-being were excluded from the model.

The second stepwise multiple regression was run with PSWQ scores collected 3 months after the end of the lockdown (T2). The final model was significant, [*F*_(3,136)_ = 34.372, *p* < 0.001], and explained 43.1% of variance in the level of worry [*R*^2^ = 0.431, Adjusted *R*^2^ = 0.419, Δ*F*_(1,136)_ = 5.45, *p* < 0.021]. Initial higher agreeableness levels and dysfunctional coping strategies predicted increases in worry from T0 to T2, after controlling for initial worry levels and socio-demographic data. Specifically, dysfunctional coping strategies showed a significant positive effect (β = 0.247, *p* = 0.001), whereas agreeableness showed a significant negative effect (β = −0.156, *p* = 0.021). Moreover, dysfunctional coping strategies alone explained 5% of the variance in worry levels, so a wide use of dysfunctional coping strategies predicted high levels of worry at T2 (*t* = 3.533, *p* = 0.001). Emotion- and problem-focused coping, extraversion, neuroticism, openness, conscientiousness, and psychological well-being were excluded from the model.

**FIGURE 1:**
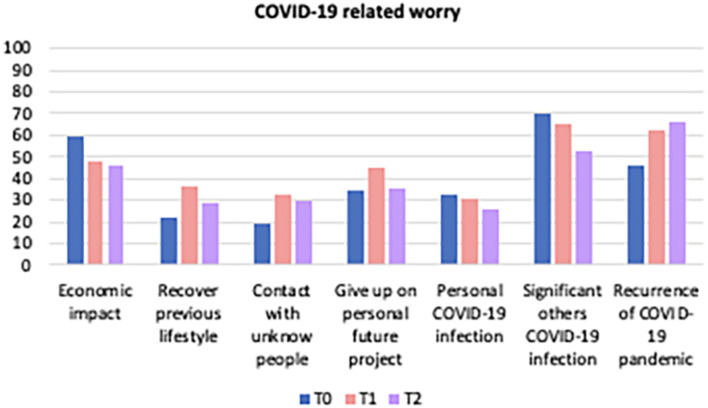
COVID-19 related worry.

Also worry related to specific COVID-19 areas/factors was recorded (e.g., risks for personal future projectuality, economic impact). These were collected by 3-point scales not to respond to specific research hypotheses but only to report anecdotally on the sample. While inferential value in respect to the population could not be attributed to these data, it is interesting to report them as an example of the COVID-19 scenario with mere descriptive value. Figure 1 shows the percentage of participants with elevated COVID-19 related worry during the three times of evaluations. During that time, there was only a linear increase of subjects who reported high levels of worry related to the recurrence of COVID-19 pandemic in the future. Additional descriptive analyses revealed that 3 months after the end of lockdown, participants with moderate/high PSWQ scores (based on the cut offs used in the literature, see Meyer et al., 1990) reported to be highly worried about outbreak economic impact (60.5%), giving up on personal future projects (44.2%), significant others COVID-19 infection (58.1%) and the recurrence of COVID-19 pandemic (67.4%). The descriptive analysis also tried to see what people with specific personality characteristics, which were found to be significant from the regression analyzes, worry more about. Based on quartiles of the personality scales, in line with Al Moubayed et al. (2014), results showed that individuals with scores in the top quartile (75%) of agreeableness reported high levels of specific COVID-19 related worries for the economic impact (*M* = 2.28 out of 3; *SD* = 0.86), risk of significant others’ COVID-19 infection (*M* = 2.35 out of 3; *SD* = 0.76) and recurrence of COVID-19 (*M* = 2.52 out of 3; *SD* = 0.73). Finally, scores in the top quartile of neuroticism correspond to higher levels of specific COVID-19 related worries for recurrence of COVID-19 emergency (*M* = 2.68 out of 3; *SD* = 0.54) and significant other COVID-19 infection (*M* = 2.47 out of 3; *SD* = 0.73).

## Discussion

This prospective longitudinal study aimed at briefly documenting the psychological impact of the first wave of COVID-19 in the Italian context, in terms of worry and its impact on psychological well-being especially. COVID-19 represents an unprecedented threat to mental health and a psychological challenge, specifically in world countries that have been strongly affected by the pandemic and the consequent restrictive measures adopted. In comparison with other longitudinal studies that focused the attention on the psychological impact of the COVID-19 lockdown and its increased signs of psychological suffering (Roma et al., 2020; Salfi et al., 2020), we focused our attention on a wider period, to assess the prevalence of any psychological symptoms even 3 months after the end of the lockdown. Our findings have shown that the levels of worry significantly decreased throughout the first wave caused by COVID-19 in Italy, while the levels of psychological well-being significantly increased. Other studies emphasized changes not only in emotions but also in health behavior along the COVID-19 phases, for example Cecchetto et al. (2021) found that negative emotions experienced during the initial phase of the lockdown influenced eating behavior leading to more frequent binge eating. Furthermore, they found a significant reduction in emotional eating and binge eating related to a decrease of the negative emotions between the onset of lockdown and the second phase of the COVID-19 pandemic. This example shows that the emotions and mood felt during the first phase of the lockdown could affect health conduct and citizen’s quality of life. Several factors may contribute to explain the trend emerging from multiple studies; in particular, it could be related to individuals’ progressive acquisition of the ability to cope with stressful events, as suggested by Zacher and Rudolph (2021) in a longitudinal study in a German sample. Furthermore, the mitigation of the containment measures, the possibility of being able to slowly come back to the previous rhythm of life, resume contacts and movements combined with more positive/hopeful media communications may have contributed to improving the psychological state 6 months after the beginning of the first lockdown. Additionally, we observed a significant decrease in the perception of controllability concerning the spread of the virus; this may be related to the confusion generated by the context and conflicting mass media communication. It is important to underline how this aspect coexists with the improvement of emotional well-being. Some studies showed that the perception of controllability predicted the intention and the compliance with the recommended preventive measures against coronavirus infection (Sobkow et al., 2020); in addition, it may act as a protective factor for psychological health during the outbreak (Zheng et al., 2020; Petrocchi et al., 2021). As the individual perception of capacity to handle the environment was associated with the perception regarding a threat (Witt et al., 2005), it might be useful to monitor the evolution of the perception of COVID-19 diffusion’s controllability in the general population and develop interventions aimed at increasing it, to promote individual health behaviors (Brivio et al., 2020).

Our results suggest that several factors, such as personality traits and dysfunctional coping strategies, may contribute to predict worry during the first wave of COVID-19 in the Italian context. In particular, subjects with neurotic personality seem to be at greater risk of higher worry levels. Neurotic people are typically more likely to experience and report negative emotions, also in COVID-19 pandemic context as confirmed by other studies (Rettew et al., 2021). Indeed, Aschwanden et al. (2021) showed that high neuroticism levels were associated with more COVID-related concern, and worry related to the pandemic duration. It is possible that the neurotic tendency to experience negative mood was exacerbated during the solitude and isolation of the lockdown, reducing people’s ability to recover hope and an optimistic attitude in the post-lockdown phases. On the other hand, 3 months after the end of the lockdown, also the agreeableness trait emerged as a protective factor against worry levels. Agreeable people tend to have more resources for social support (Barańczuk, 2019; Yu et al., 2020) which contribute to reducing worry by means of positive social interactions and shared meaning-making regarding distressing events (Zysberg and Zisberg, 2020; Al-Omiri et al., 2021). The fact that such a protective factor emerged in the third phase specifically may be related to the renovated opportunities for social aggregation after the limitations imposed by the lockdown.

Therefore, even in the context of the pandemic, it is important to recognize the role of individual differences (Kroencke et al., 2020; Modersitzki et al., 2020; Somma et al., 2020; Osimo et al., 2021). Regarding dysfunctional coping strategies, our study confirmed that the use of these strategies was maladaptive, not only because they were correlated to worry levels as found in our previous study (Sebri et al., 2021), but also because they predicted the increase of the level of worry 3 months after the end of the lockdown. Moreover, in another study related to the present longitudinal study, it was found that dysfunctional coping strategies at the initial stages of COVID-19 increased the levels of worry which in turn mediated the relationship between the aforementioned coping strategies and state anxiety enhancing it (Cincidda et al., 2021). These results suggested the importance of taking into account both personality characteristics and dysfunctional coping strategies implemented during the pandemic in order to plan personalized interventions based on these characteristics.

Finally, even if our study showed a significant negative correlation between worry and psychological well-being, confirming the results of previous studies (Taylor et al., 2020; Cincidda et al., 2021; Sebri et al., 2021), it emerged that worry levels during the lockdown and at the end of the lockdown did not predict well-being levels 3 months after the end of the lockdown, and vice versa. What turns out to be a predictor of levels of psychological well-being is well-being itself, measured at baseline and at the end of the lockdown. Several studies have shown the role of worry in the genesis of depressive/anxiety disorders (Olatunji et al., 2010; Spinhoven et al., 2017; Prete et al., 2020) so maybe different constructs and questionnaires could be used in future studies in order to analyze the longitudinal impact of high worry levels during COVID-19 pandemic. Specifically, future studies could analyze the predictive role of worry during COVID-19 on the development of mood or anxiety disorders, instead of evaluating the impact on a variable as broad as well-being, which can be influenced by several other different parameters. In this line, while the present study aimed at analyzing the impact of worry on well-being, future studies may explore other antecedents of well-being to provide further evidence about the effects of the COVID-19 pandemic on healthy populations (Shanahan et al., 2020; Götmann and Bechtoldt, 2021).

The present study has several limitations and should be interpreted with caution. The first limitation of the study concerns the sample size, which is limited and reduced in the three evaluation times; we could not follow up with the majority of our participants, probably due to the modality of communication, the email, that limited the contact with the respondents. Furthermore, the survey relied on voluntary sampling, so the sample could be composed of highly motivated subjects to participate in the study and it may inflate the generalizability of the results. Therefore, our study should be affected by an attrition bias. The limited sample size made it impossible to run more complex analyses featuring mediating or moderating factors. However, to our knowledge, this is the first web-based longitudinal study on the psychological impact during the first wave of COVID-19 in Italian context. The second limitation concerns the measures applied in the study. In an attempt to reduce participant’s compilation time, to maintain an acceptable engagement in the study and to avoid increasing respondents’ psychological burden, we carefully balanced the number of questions and selected the short version of some of the measures, such as the Big Five Inventory–Short Form (BFI-S), that is composed of only 10 items. So, some of the selected measures are not the most sophisticated and may be more prone to measurement errors. Finally, descriptive analyses with a mere anecdotal value showed that there may be differences in worry among citizens depending on personality and specific worrying factors. Future research may consider exploring this suggestion with more sophisticated variables and dedicated methodology.

It could be interesting to conduct a further examination to gather information on changes in psychological outcomes in the different phases and waves related to COVID-19, not only in the Italian context but also in other countries heavily affected by the health emergency. Future studies should use more sophisticated trait measures, specifically about personality characteristics, to verify and confirm its relevance in terms of moderating the psychological response to the threat of COVID-19 pandemic. Furthermore, specific longitudinal studies about worry and distress should be implemented for specific categories of subjects, such as ones tested positive for COVID-19 or health professionals, higher impacted by this challenge. Finally, future studies could explore how COVID-related worry may be associated with individuals’ well-being; in particular, after controlling the well-being level at the baseline, it could be interesting to analyse whether COVID-related worry may predict well-being across time or vice versa. Despite this limitation, the strength of our study is the longitudinal nature of the work that extends the information on the “tracking” of worry and emotional well-being of Italian citizens over the first wave of the COVID-19 pandemic.

In conclusion, our research suggests the presence of risk factors for the development of worry, detectable in personality characteristics and dysfunctional coping strategies. Furthermore, our findings highlight a long-term reduction in both levels of worry and in the perception of controllability of the virus diffusion, which appear to be linear and linked to the easing of COVID-19 restrictions and the improvement of cases’ infection conditions; our results showed an increased in psychological well-being levels. However, worry did not predict psychological well-being during the first wave of COVID-19 in an Italian sample.

Based on these findings, mental health policymakers should design tailored interventions able to improve the perception of virus diffusion management, as well as to address the psychological needs of Italian citizens and to support it, including a plan for the follow-up evaluation. In addition, in order to improve public health communication and the effectiveness of interventions, it is necessary to highlight individual differences among people, with a special focus on personality characteristics, specifically to people with high levels of neuroticism and with openness to experience, who resulted at higher risk of worry and distress. As suggested by Triberti et al. (2021), citizens’ personality characteristics should be considered by public health communication to improve the effectiveness of the messages and to promote positive behavioral changes related to COVID-19 pandemic. In particular, it might be useful to emphasize positive consequences for one’s life in following messages and, for more open individuals, to emphasize the possibility to find more unconventional ways to adjust to the pandemic and to use their high curiosity toward new things as a way to better cope with the new situation. In addition, evidence-based interventions (such as Cognitive Behavioral Therapy) could be devised to reduce the levels of worry that people experienced during the early stages of lockdown and to decrease the use of dysfunctional coping strategies by providing alternative and more functional coping strategies. In an uncertain situation, web-based mindfulness training or relaxation ones (Spinhoven et al., 2017; Mauri et al., 2018; Pizzoli et al., 2020; Antonova et al., 2021) could be planned to reduce worries, negative thoughts, and expectations, and increase psychological well-being.

## Authors’ Note

GO, CC, VS, and LS are Ph.D. students within the European School of Molecular Medicine (SEMM).

## Data Availability Statement

The raw data supporting the conclusions of this article will be made available by the authors, without undue reservation.

## Ethics Statement

The studies involving human participants were reviewed and approved by the Institutional Review Board (IRB) of the University of Milan. The patients/participants provided their written informed consent to participate in this study.

## Author Contributions

GO, CC, and VS conceptualized the ideas presented in the article and wrote the first draft. GO, CC, VS, and LS collected the data. GO, CC, and ST performed the analysis. GO, ST, and LS edited the manuscript. RF, BP, and BD’O contributed equally to revision and edited the manuscript. RF, BP, BD’O, and GP contributed with important intellectual content. GP supervised the whole process. All authors provided feedback and approved the final version of the manuscript.

## Conflict of Interest

The authors declare that the research was conducted in the absence of any commercial or financial relationships that could be construed as a potential conflict of interest.

## Publisher’s Note

All claims expressed in this article are solely those of the authors and do not necessarily represent those of their affiliated organizations, or those of the publisher, the editors and the reviewers. Any product that may be evaluated in this article, or claim that may be made by its manufacturer, is not guaranteed or endorsed by the publisher.
